# Refractory Ventricular Fibrillation Associated With a Coronary Vasospasm and Fixed Moderate Coronary Stenosis

**DOI:** 10.1016/j.jaccas.2026.109104

**Published:** 2026-07-01

**Authors:** Hirokazu Nishido, Satoshi Honda, Teruo Noguchi

**Affiliations:** Department of Cardiovascular Medicine, National Cerebral and Cardiovascular Center, Osaka, Japan

**Keywords:** acute coronary syndrome, cardiovascular disease, imaging, ventricular fibrillation

## Abstract

**Background:**

A coronary vasospasm is an important but under-recognized cause of ventricular fibrillation, particularly when angiography shows a nonocclusive fixed coronary lesion.

**Case Summary:**

A previously healthy 36-year-old man presented with recurrent early-morning chest pain followed by refractory ventricular fibrillation requiring venoarterial extracorporeal membrane oxygenation. Coronary angiography showed moderate proximal left anterior descending artery stenosis with functionally significant ischemia. However, optical coherence tomography revealed no plaque rupture or thrombus. Because the coronary anatomy was discordant with the severity of presentation, acetylcholine provocation testing was performed under circulatory support and confirmed a severe coronary vasospasm.

**Discussion:**

This case highlights the importance of a mechanism-based evaluation in survivors of malignant ventricular arrhythmias.

**Take-Home Messages:**

When fixed coronary findings do not fully explain the clinical presentation, a coronary vasospasm should be considered. In selected high-risk patients, carefully performed pharmacologic provocation testing under the appropriate hemodynamic support may be essential for establishing the diagnosis.

## Visual Summary

**Figure:**
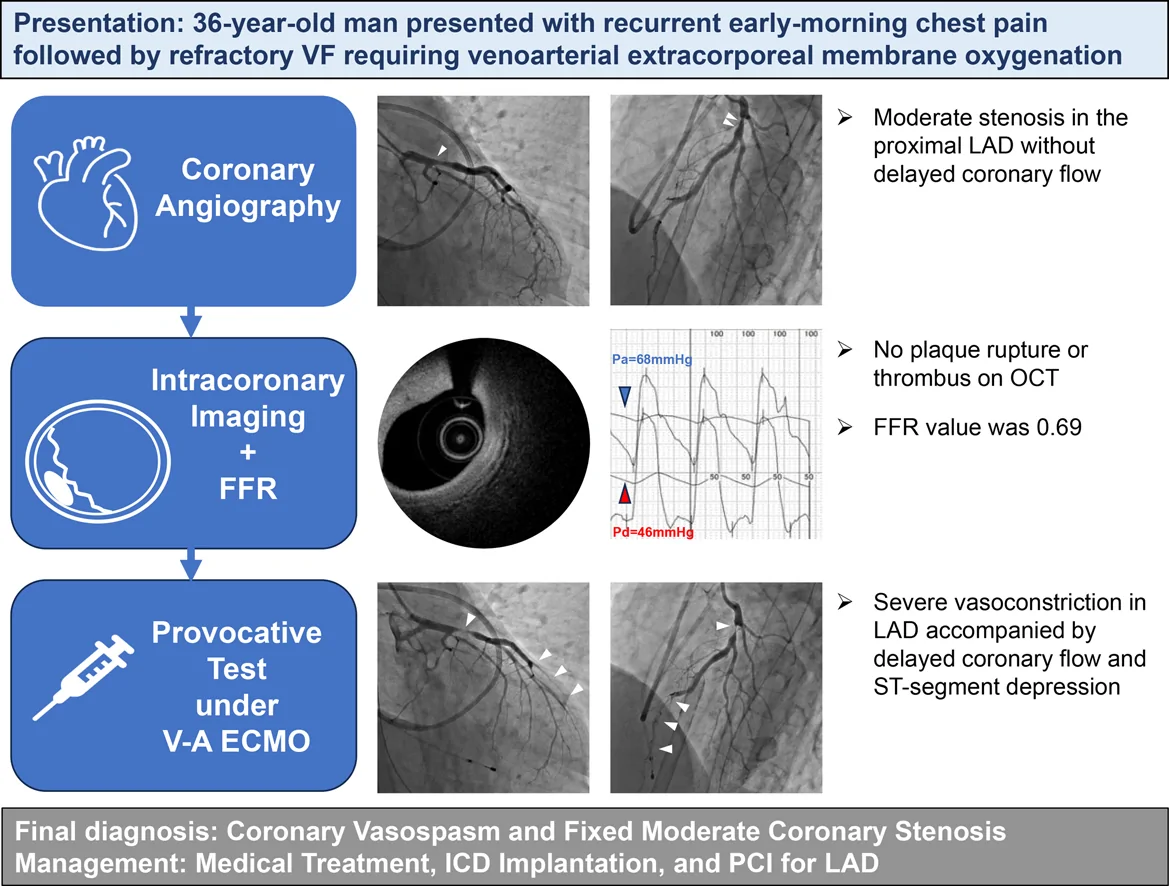

**Visual Summary:**
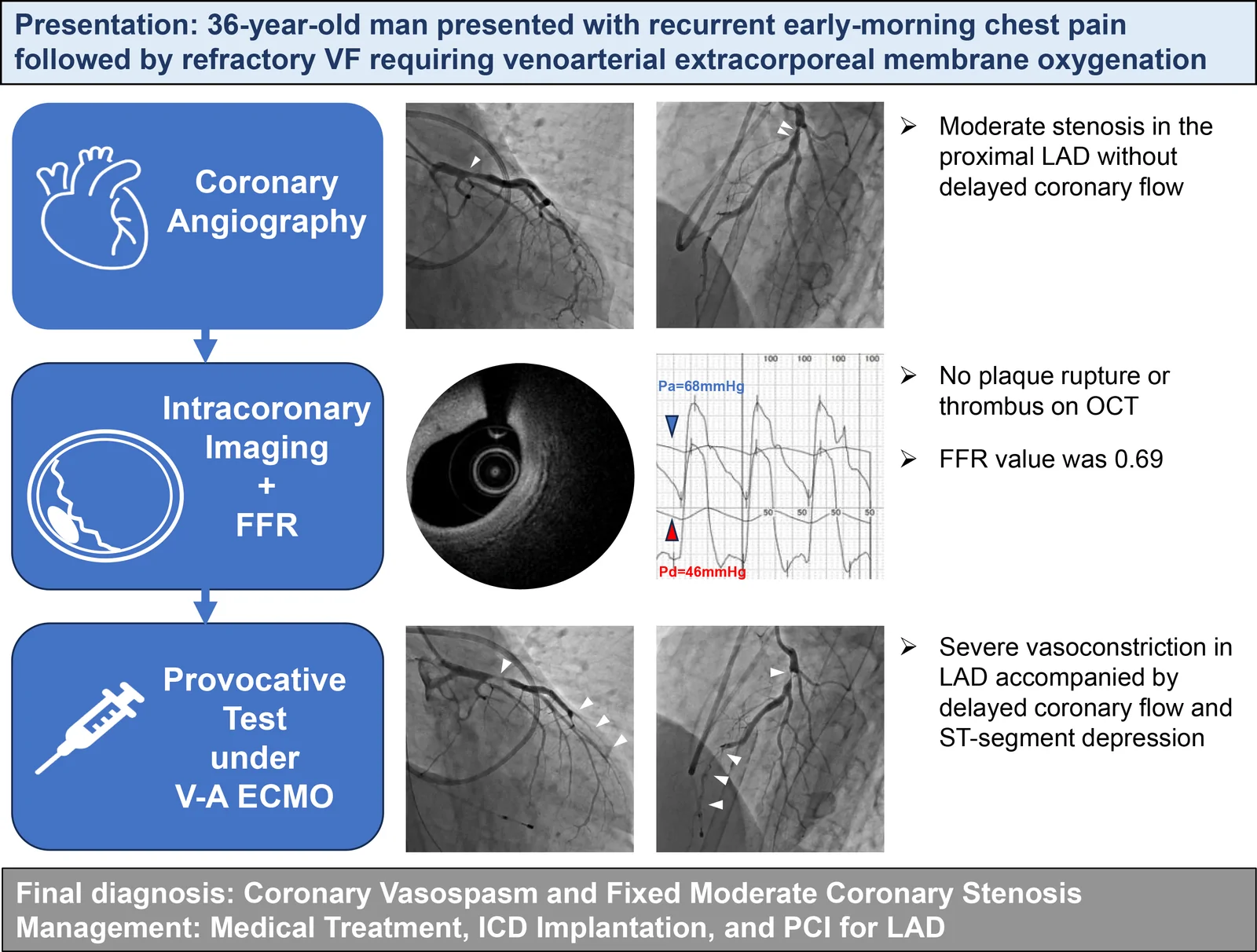
Mechanism-Based Diagnosis of Refractory Ventricular Fibrillation FFR = fractional flow reserve; ICD = implantable cardioverter-defibrillator; LAD = left anterior descending; OCT = optical coherence tomography; PCI = percutaneous coronary intervention; VA-ECMO = venoarterial extracorporeal membrane oxygenation; VF = ventricular fibrillation.

## History of Presentation

A previously healthy 36-year-old man presented with a 2-week history of recurrent early-morning chest tightness. On the day of admission, he developed chest discomfort triggered by defecation and collapsed shortly thereafter. Emergency medical services documented ventricular fibrillation (VF). Despite multiple defibrillation attempts, return of spontaneous circulation was not achieved in the field. At the receiving hospital, persistent VF prompted emergent initiation of venoarterial extracorporeal membrane oxygenation (VA-ECMO), resulting in return of spontaneous circulation after a low-flow time of 16 minutes.

## Past Medical History

He had no significant past medical history. He reported a family history of myocardial infarction, though not of premature onset.

## Differential Diagnosis

The recurrent early-morning symptoms strongly suggested a coronary vasospasm as the primary trigger for VF. Given the patient’s young age, inherited arrhythmia syndromes, such as Brugada or long QT syndrome, and cardiomyopathies remained essential considerations.

## Investigations

Emergent coronary angiography revealed moderate stenosis in the proximal left anterior descending (LAD) artery. However, distal coronary flow was preserved, and the angiographic severity of the lesion was not considered sufficient to account for the refractory VF; therefore, conservative management was initially selected.

**Figure 1:**
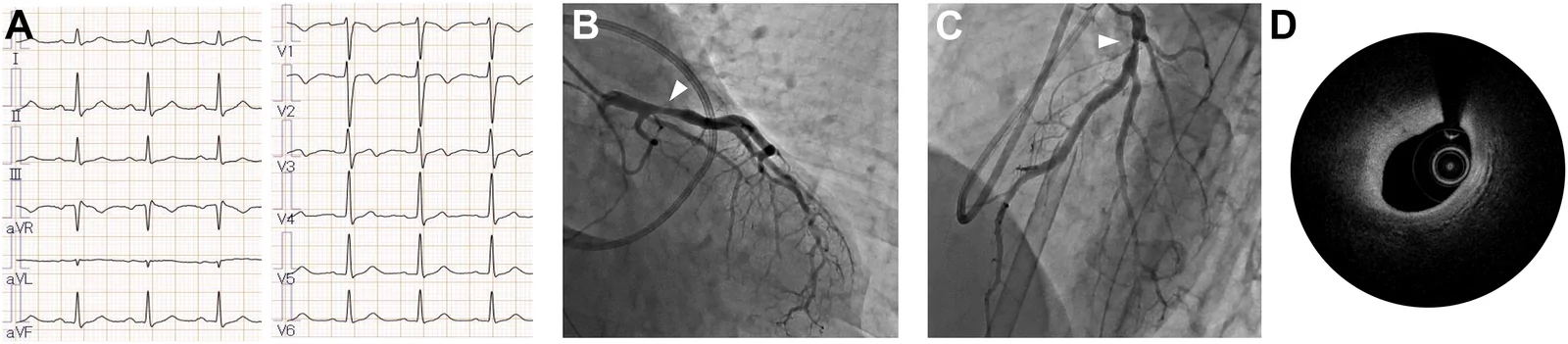
Initial Evaluation by Coronary Angiography and Intravascular Imaging (A) Electrocardiography showed a corrected QT interval of 433 ms with no ST-segment changes. (B and C) Coronary angiography demonstrated moderate stenosis in the proximal left anterior descending artery (white head). (D) Optical coherence tomography demonstrated the absence of a layered plaque characteristic of vasospastic angina. A lipid-rich plaque with a thick fibrous cap was observed.

On day 3, the patient was transferred to our tertiary center for further evaluation. Hemodynamics were stable under VA-ECMO support, and no recurrent ventricular arrhythmias occurred. Electrocardiography demonstrated terminal T-wave inversion in leads V_1_–V_3_ without ST-segment deviation or QT prolongation (Figure 1A). Transthoracic echocardiography showed normal left ventricular systolic function without structural abnormalities.

Repeat coronary angiography on day 4 confirmed unchanged moderate proximal LAD artery stenosis (Figure 1B and 1C, Video 1, Video 2). Optical coherence tomography demonstrated a lipid-rich plaque without evidence of plaque rupture or intracoronary thrombus. Fractional flow reserve (FFR) measured 0.69, indicating physiologically significant ischemia (Figure 1D, Video 3). However, the overall findings were discordant with a rupture-mediated acute coronary syndrome, which could explain the malignant arrhythmia.

**Figure 2:**
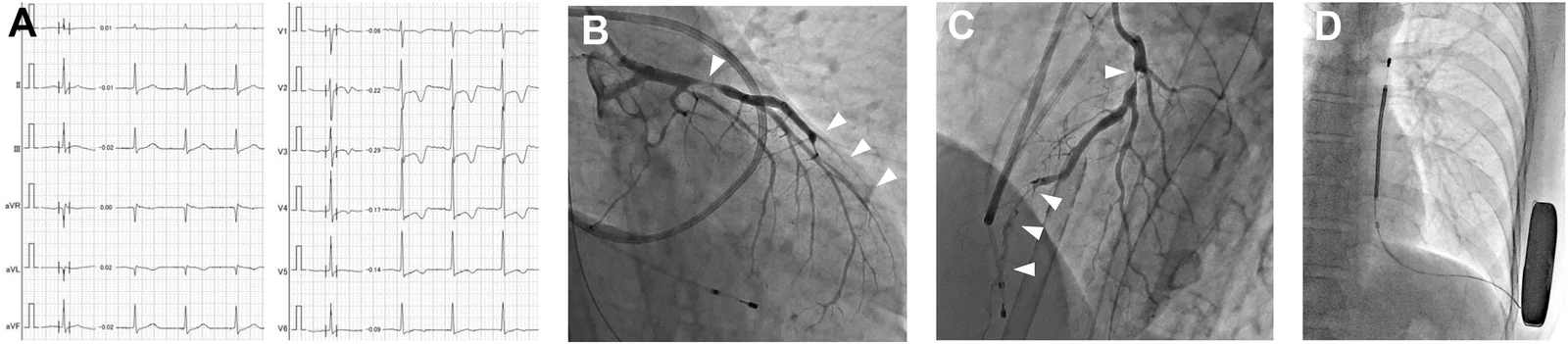
Acetylcholine Provocation Test and Subcutaneous Implantable Cardioverter-Defibrillator Implantation (A) Administration of acetylcholine (20 μg) induced horizontal ST-segment depression of up to 2 mm in leads V_2_-V_4_. (B and C) Administration of acetylcholine (20 μg) induced severe stenosis in the proximal and distal segments of the left anterior descending artery (white heads). (D) The patient had an out-of-hospital cardiac arrest due to ventricular fibrillation associated with coronary vasospastic angina and was treated with insertion of an implantable cardioverter-defibrillator. Abbreviations as in Figure 1.

Pharmacologic provocation testing with intracoronary acetylcholine was performed under close hemodynamic monitoring with VA-ECMO support. Administration of acetylcholine induced severe vasoconstriction in both proximal and distal segments of the LAD artery, accompanied by delayed coronary flow and transient ST-segment depression, confirming the diagnosis of a coronary vasospasm (Figure 2A to 2C, Video 4, Video 5).

## Management

Calcium-channel blocker therapy was initiated, resulting in complete resolution of anginal symptoms. The patient was successfully weaned from VA-ECMO on day 4. After full neurologic recovery, an implantable cardioverter-defibrillator (ICD) was inserted for secondary prevention, given the history of vasospasm-induced VF (Figure 2D). The decision to proceed with percutaneous coronary intervention (PCI) required explicit consideration because vasospasm, rather than plaque rupture, appeared to be the dominant trigger of VF. However, the LAD artery lesion was not trivial: FFR was 0.69, indicating physiologically significant fixed ischemia, and the lesion could plausibly amplify vasospasm-related flow limitation. In this setting, we considered the fixed stenosis to be a substrate that may have lowered the threshold for severe ischemia during a spasm. Therefore, after initiation of vasodilator therapy and stabilization of the acute phase, staged PCI of the proximal LAD artery was performed as adjunctive treatment.

## Outcome and Follow-Up

The patient was successfully weaned from VA-ECMO on day 4. He was discharged in stable condition and remained free from recurrent chest pain or ventricular arrhythmias during follow-up.

## Discussion

Out-of-hospital cardiac arrest due to refractory VF in the presence of both coronary vasospasm and fixed coronary stenosis presents important diagnostic and therapeutic challenges.^1^^,^^2^ In this case, our approach—integrating coronary angiography and intracoronary imaging to assess plaque morphology, physiological assessment, and carefully performed pharmacologic provocation testing with attention to procedural safety—provided critical insights into both diagnosis and long-term management.

An important aspect of this case is that a coronary vasospasm was definitively identified by acetylcholine provocation testing in a high-risk patient under circulatory support.

Acetylcholine or ergonovine provocation testing is the gold standard for diagnosing a coronary vasospasm.^1^^,^^3^^,^^4^ However, in patients with significant coronary stenosis or in the acute phase after cardiac arrest, such testing may induce severe ischemia or malignant arrhythmias.^5^ Therefore, it should be performed under careful hemodynamic monitoring with immediate access to vasodilators, defibrillation, and circulatory support. In our case, VA-ECMO provided circulatory support that allowed testing in a controlled setting and helped identify a coronary spasm as the likely trigger for VF. This diagnostic precision is clinically important because the treatment strategies differ according to the dominant mechanism: PCI is important for fixed stenosis, whereas medical therapy centered on calcium channel blockers is essential for preventing recurrent vasospastic episodes.^6^ Accordingly, in selected high-risk patients, careful evaluation under appropriate support may be necessary to distinguish dynamic vasospasm from fixed ischemic substrates.

Regarding secondary prevention, the decision to implant an ICD was justified by the severe clinical risk profile.^1^^,^^2^ Although calcium channel blockers are the cornerstone of vasospasm treatment, survivors of out-of-hospital cardiac arrest with vasospasm-induced VF remain at high risk of recurrent lethal events despite optimal medical therapy. Current guideline recommendations support consideration of ICD insertion for survivors of sudden cardiac death related to a coronary vasospasm.^1^ In this case, the patient experienced refractory VF, and ICD insertion was therefore considered appropriate for secondary prevention.

Although a vasospasm was considered the dominant trigger of VF, PCI for the proximal LAD artery lesion was also considered. FFR demonstrated physiologically significant ischemia, suggesting that the organic coronary stenosis itself could increase the future risk of cardiovascular events.^7^ In addition, the fixed stenosis may have amplified ischemic vulnerability and contributed to malignant arrhythmia in the setting of superimposed spasm. Therefore, after initiation of antispasm therapy and stabilization of the acute phase, PCI of the proximal LAD artery was performed as adjunctive treatment.

## Conclusions

This case was initially presumed to represent VF secondary to myocardial infarction. However, neither angiographic stenosis nor physiologic assessment with FFR adequately explained the malignant arrhythmia. Intravascular imaging excluded plaque rupture, and acetylcholine provocation testing, performed safely in a controlled setting under V-A ECMO support during the acute phase, unmasked severe coronary vasospasm as the arrhythmogenic substrate.

This case highlights the critical importance of comprehensive, multimodal ischemic assessment in survivors of sudden cardiac arrest. When anatomical and physiological findings are discordant with the clinical presentation, dynamic coronary pathology must be considered. Early and systematic evaluation—including pharmacologic provocation under appropriate hemodynamic support—can establish the diagnosis, guide targeted therapy, and improve clinical outcomes.

## Funding support and author disclosures

This work was supported by 10.13039/501100001691JSPS KAKENHI grant number 23K15116. The authors have reported that they have no relationships relevant to the contents of this paper to disclose.

**Take-Home Messages**

• When the clinical presentation of ventricular fibrillation is disproportionate to angiographic findings, a coronary vasospasm should be actively considered, even in the presence of fixed coronary stenosis.
• Careful provocation testing under appropriate hemodynamic support may help identify the dominant ischemic mechanism and guide complementary therapy.

## Appendix

Video 1 Coronary angiography demonstrated moderate stenosis in the proximal left anterior descending artery in the right anterior oblique caudal view.

Video 2 Coronary angiography demonstrated moderate stenosis in the proximal left anterior descending artery in the left anterior oblique cranial view.

Video 3 Optical Coherence Tomography Imaging of the Target Coronary Artery Demonstrates a Lipid-Rich Plaque With a Thick Fibrous Cap

Video 4 Administration of acetylcholine (20 μg) induced severe stenosis in the proximal and distal segments of the left anterior descending artery in the right anterior oblique caudal view.

Video 5 Administration of acetylcholine (20 μg) induced severe stenosis in the proximal and distal segments of the left anterior descending artery in the left anterior oblique cranial view.

## References

[1] Al-Khatib S.M., Stevenson W.G., Ackerman M.J. (2018). 2017 AHA/ACC/HRS guideline for management of patients with ventricular arrhythmias and the prevention of sudden cardiac death. J Am Coll Cardiol.

[2] Zeppenfeld K., Tfelt-Hansen J., de Riva M. (2022). 2022 ESC guidelines for the management of patients with ventricular arrhythmias and the prevention of sudden cardiac death. Eur Heart J.

[3] Rao S.V., O'Donoghue M.L., Ruel M. (2025). 2025 ACC/AHA/ACEP/NAEMSP/SCAI guideline for the management of patients with acute coronary syndromes: a report of the American College of Cardiology/American Heart Association Joint Committee on clinical practice guidelines. J Am Coll Cardiol.

[4] JCS Joint Working Group (2014). JCS 2013 guideline on diagnosis and treatment of vasospastic angina (coronary spastic angina). Circ J.

[5] Kim H.L., Kim M.A., Park J.B. (2024). Coronary vasospasm presenting in a catastrophic way. JACC Case Rep.

[6] Virani S.S., Newby L.K., Arnold S.V. (2023). 2023 AHA/ACC/ACCP/ASPC/NLA/PCNA guideline for the management of patients with chronic coronary disease: a report of the American Heart Association/American College of Cardiology Joint Committee on clinical practice guidelines. J Am Coll Cardiol.

[7] Barbato E., Toth G.G., Johnson N.P. (2014). Prognostic value of fractional flow reserve: linking physiologic severity to clinical outcomes. J Am Coll Cardiol.

